# Supplementary figures and images for: Molecular epidemiology of Leptospira spp. among wild mammals and a dog in Amami Oshima Island, Japan
Source: PLoS One. 2021 Apr 22;16(4):e0249987. doi: 10.1371/journal.pone.0249987 (PMC8061989; doi:10.1371/journal.pone.0249987)

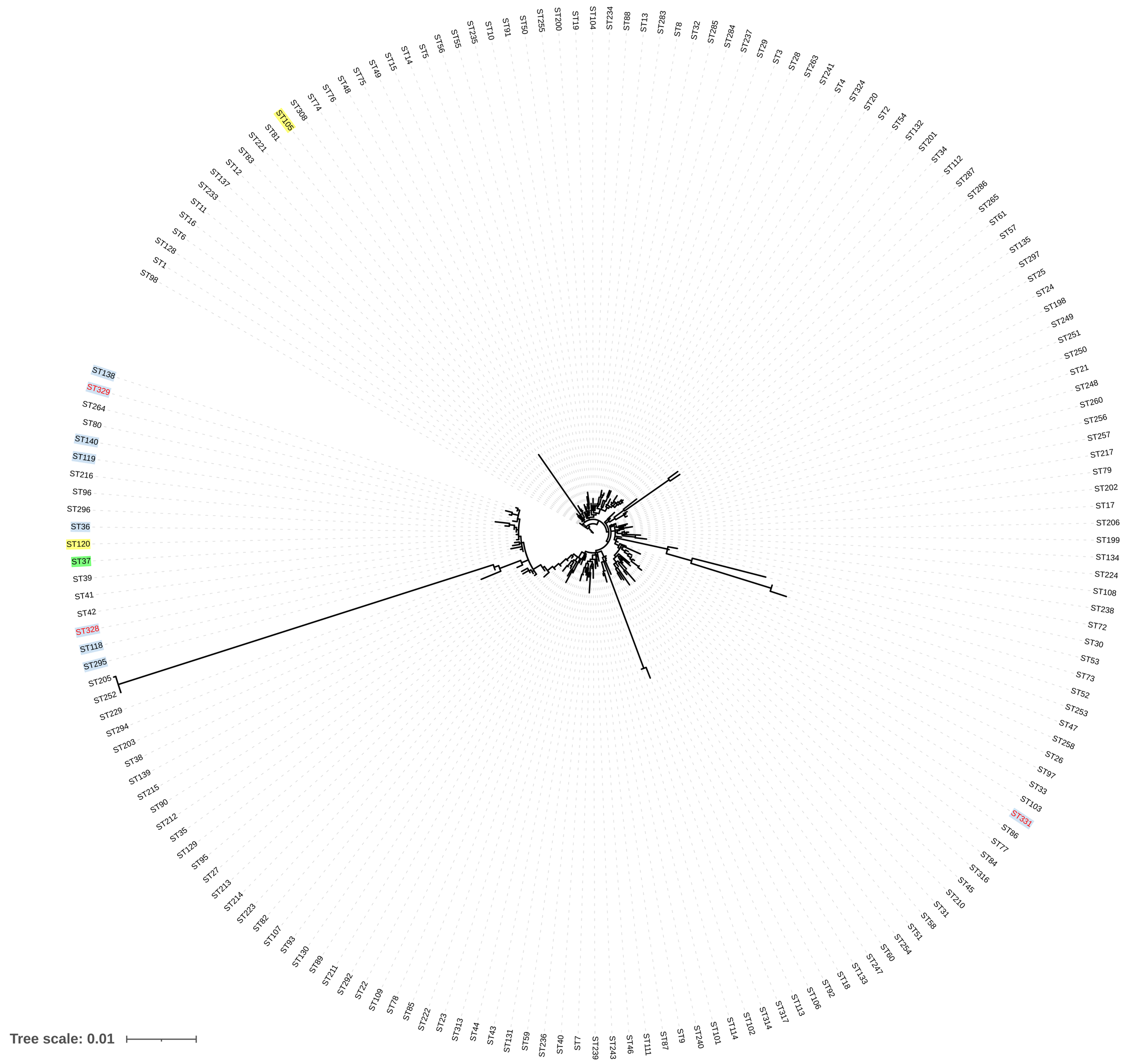

Supplement: S1 Fig — The STs of serogroups Australis and Hebdomadis identified in Japan are highlighted in yellow and blue, respectively. The ST highlighted in green indicates that it is identified both in the serogroups Australis and Hebdomadis in Japan. The novel STs identified in this study are indicated in red font. (TIF) [file pone.0249987.s001.tif]
