# Supplementary material for: Molecular epidemiology of Leptospira spp. among wild mammals and a dog in Amami Oshima Island, Japan
Source: PLoS One. 2021 Apr 22;16(4):e0249987. doi: 10.1371/journal.pone.0249987 (PMC8061989; doi:10.1371/journal.pone.0249987)
Supplement: S1 Table — (DOCX) [file pone.0249987.s002.docx]

| S1 Table. Clinical characteristics and laboratory data of D-KS19-7K | | | | | |
| --- | --- | --- | --- | --- | --- |
| Symptom | | |  | Biochemistry | |
| Vomming | | |  | ALT | 105 U/l |
| Hyperemia and hemorrhage of the mucous membranes | | |  | ALP | 1784 U/l |
| Jaundice | | |  | T-Bil | 14.3 mg/dl |
|  |  |  |  | BUN | >140 mg/dl |
| Complete blood count | |  |  | Cre | 12.0 mg/dl |
| WBC | 29.1×10^3^/μl |  |  | Glu | 118 mg/dl |
| RBC | 5.0×10^6^/μl |  |  | Na | 140 mmol/l |
| Hb | 12.3 g/dl |  |  | K | 6.1 mmol/l |
| Ht | 34.5% |  |  | Cl | 98 mmol/l |
| Plt | 33×10^3^/μl |  |  |  |  |
