# Supplementary material for: Molecular epidemiology of Leptospira spp. among wild mammals and a dog in Amami Oshima Island, Japan
Source: PLoS One. 2021 Apr 22;16(4):e0249987. doi: 10.1371/journal.pone.0249987 (PMC8061989; doi:10.1371/journal.pone.0249987)
Supplement: S2 Table — (DOCX) [file pone.0249987.s003.docx]

| S2 Table. Clinical characteristics and laboratory data of the female hunting dog confirmed as leptospirosis in Amami Oshima Island in 2018 | | | | | |
| --- | --- | --- | --- | --- | --- |
| Symptom | | |  | Biochemistry | |
| Vomming | | |  | ALT | 100 U/l |
| Hyperemia and hemorrhage of the mucous membranes | | |  | ALP | 218 U/l |
| Jaundice | | |  | T-Bil | 1.2 mg/dl |
|  |  |  |  | TP | 7.0 g/dl |
| Complete blood count | |  |  | ALB | 3.1 g/dl |
| WBC | 23.9×10^3^/μl |  |  | BUN | 88 mg/dl |
| RBC | 7.1×10^6^/μl |  |  | Cre | 4.5 mg/dl |
| Hb | 16.6 g/dl |  |  | Glu | 107 mg/dl |
| Ht | 44% |  |  | Na | 144 mmol/l |
| Plt | 120×10^3^/μl |  |  | K | 4.1 mmol/l |
|  |  |  |  | Cl | 100 mmol/l |
| MAT^a^ |  |  |  |  |  |
| serovars Hebdomadis and Kremastos | | |  |  |  |
| Acute | <50 |  |  |  |  |
| Convalescent | 800 |  |  |  |  |

^a^ Microscopic agglutination test
